# Spatial analysis of malaria in Anhui province, China

**DOI:** 10.1186/1475-2875-7-206

**Published:** 2008-10-10

**Authors:** Wenyi Zhang, Liping Wang, Liqun Fang, Jiaqi Ma, Youfu Xu, Jiafu Jiang, Fengming Hui, Jianjun Wang, Song Liang, Hong Yang, Wuchun Cao

**Affiliations:** 1 Beijing Institute of Microbiology and Epidemiology, State Key Laboratory of Pathogen and Biosecurity, Beijing, PR China; 2 Center for Public Health Information, National Center for Disease Control and Prevention, Beijing, PR China; 3 Anhui Center for Disease Control and Prevention, Hefei, PR China; 4 College of Public Health, The Ohio State University, Columbus, Ohio, USA

## Abstract

**Background:**

Malaria has re-emerged in Anhui Province, China, and this province was the most seriously affected by malaria during 2005–2006. It is necessary to understand the spatial distribution of malaria cases and to identify highly endemic areas for future public health planning and resource allocation in Anhui Province.

**Methods:**

The annual average incidence at the county level was calculated using malaria cases reported between 2000 and 2006 in Anhui Province. GIS-based spatial analyses were conducted to detect spatial distribution and clustering of malaria incidence at the county level.

**Results:**

The spatial distribution of malaria cases in Anhui Province from 2000 to 2006 was mapped at the county level to show crude incidence, excess hazard and spatial smoothed incidence. Spatial cluster analysis suggested 10 and 24 counties were at increased risk for malaria (*P *< 0.001) with the maximum spatial cluster sizes at < 50% and < 25% of the total population, respectively.

**Conclusion:**

The application of GIS, together with spatial statistical techniques, provide a means to quantify explicit malaria risks and to further identify environmental factors responsible for the re-emerged malaria risks. Future public health planning and resource allocation in Anhui Province should be focused on the maximum spatial cluster region.

## Background

Malaria is one of the leading causes of morbidity and mortality in the world. Indeed, more than 2.4 billion people are exposed to the risk of malaria [1]. Malaria kills between 1.1 and 2.7 million people each year [1,2]. Malaria is one of major parasitic diseases with a wide distribution in China. The prevalence gradually decreases from south to north. Southern parts of 25°NL (Nanling Mountains) used to be the hyper- or meso-endemic regions, where falciparum malaria was widely present. Meso- and hypo-endemic areas were between 25–33°NL (from Nanling to Qinling Mountains and the Huai River), where *vivax *malaria was predominant, though falciparum malaria also existed and focal outbreaks often occurred. In the region north of 33°NL (north of Qinling Mountain and the Huai River), malaria was of low endemicity and *Plasmodium vivax *was the only species present; temporary epidemics were occasionally caused by imported falciparum malaria [3].

The provinces of Yunnan and Hainan are the areas where malaria has been the most endemic with high transmission of *Plasmodium falciparum*. Since 2000, a malaria resurgence has occurred in China. In addition to the southern mountainous area of Hainan province and the border area of Yunnan province, most re-emerged malaria occurred in central China, along the Huai River. Anhui Province is the most seriously affected area in China, with the highest number of malaria cases in 2006. The incidence of malaria shows high variability at the county level. A better understanding of the spatial distribution patterns of malaria would help to identify areas and population at high risk and may better prevent and control malaria in this province.

The use of GIS with spatial statistics, including spatial smoothing and cluster analysis, has been applied to other diseases, in which it is often used to analyse and more clearly characterize the spatial patterns [4-8]. Spatial smoothing is used to reduce random variation associated with small populations and enables observation of gradients or holes in disease incidence that may not be apparent from direct observation of the raw data. Spatial cluster analysis is conducted to identify whether cases of disease are geographically clustered [9-11].

In this study, GIS-based spatial analyses involving spatial smoothing and spatial clustering analysis were conducted to characterize geographic distribution patterns of malaria cases. Spatial analysis was used to identify the distribution pattern of malaria and population at high risk at the county level. The technique corrects for multiple comparisons, adjusts for the heterogeneous population densities among the different areas, detects foci without prior specification of suspected location or size, and thereby overcomes pre-selection bias and allows for adjustment of confounders [11,12].

## Materials and methods

### Data collection and management

The study site is Anhui Province (114.85° ~119.69°E, 29.38° ~34.74°N), located in the area between the Changjiang River and Huai River, the third largest river in China. The maximum distance from east to west is about 430 km and from north to south is about 586 km. The area has a population of 58,358,232 (the fifth national census in 2000) and encompasses 139,600 square kilometers.

Records on malaria cases between 2000 and 2006 were obtained from the National Notifiable Disease Surveillance System (NNDSS). For conducting a GIS-based analysis on the spatial distribution of malaria, the county-level polygon map at 1:1,000,000 scale was obtained, on which the county-level point layer containing information regarding latitudes and longitudes of central points of each county was created. Demographic information based on the 2000 census was integrated in terms of the administrative code. All malaria cases were geo-coded and matched to the county-level layers of polygon and point by administrative code using the software ArcGIS 9.1 (ESRI Inc., Redlands, CA, USA).

### GIS mapping and smoothing

To alleviate variations of incidence in small populations and areas, annualized average incidences of malaria per 100,000 at each administrative region over the seven year period were calculated, and spatial rate smoothing was implemented. Based on annualized average incidence, all counties were grouped into four categories: non-endemic area, low endemic area with annualized average incidence between 0 and 5 per 100,000, medium endemic area with the incidence between 5 and 30 per 100,000, and high endemic area with the incidence over 30 per 100,000. The four types of counties were colour-coded on maps.

To assess the risk of malaria in each county, an excess hazard map was produced. The excess hazard represents the ratio of the observed incidence at each county over the average incidence of all endemic areas, the later was calculated by the number of cases over the total number of people at risk instead of the annualized incidence of a county [13].

The technique of incorporating data from surrounding areas in an image or map to define a new data value for the area of interest is called spatial filtering. Spatial filtering can involve smoothing or sharpening the data of interest. The spatial smoothing was performed to reduce random noise in the data that comes from the high variance characteristic of small populations or small case numbers [14]. The smoothed incidence was computed from the total number of cases in a spatial 'window' divided by the total number of people at risk within the 'window', which was specified using a spatial weights file including both county and its neighbor counties' locations. Each smoothed incidence was calculated once the 'window' core overlapped with a county center. So the first step in the analysis was to construct a spatial weights file that contained information on 'neighborhood' structure of each county. The k-nearest neighbour criterion ensured each observed object had exactly the same number (k) of neighbours. In the analysis, six neighbours were chosen for each county by k-nearest neighbour criterion. The second step was to load the weight file and carry out smoothing analysis [13].

### Cluster analysis

Spatial cluster analysis was performed on the confirmed cases of malaria to test whether the cases were distributed randomly over space and, if not, to evaluate any identified spatial disease clusters for statistical significance. 'Spatial scan statistics' was used to test the null hypothesis that the relative risk (RR) of malaria was the same between any county groups, or collection of county groups, and the remaining county groups. Areas with differing sizes were scanned without knowledge on cluster size and location to avoid selection bias. SaTScan software version 6.0 [15], designed specifically to implement this test, imposed a circular window on the map. This window moved over the area and centered on the centroid of each county group. The area within the circular window varied in size from zero to a maximum radius, never including > 50% of the total population. The SaTScan software tested for possible clusters within the variable window around the centroid of each county group. Cluster analysis was performed with the default maximum spatial cluster size of < 50% of the population and again with a smaller maximum cluster size of < 25% to look for possible sub-clusters. For each window of varying position and size, the software tested the risk of malaria within and outside the window, with the null hypothesis of equal risk. This procedure compensated for the inherent bias in multiple testing [9].

## Results

### Spatial distribution of malaria in Anhui Province

**Figure 1:**
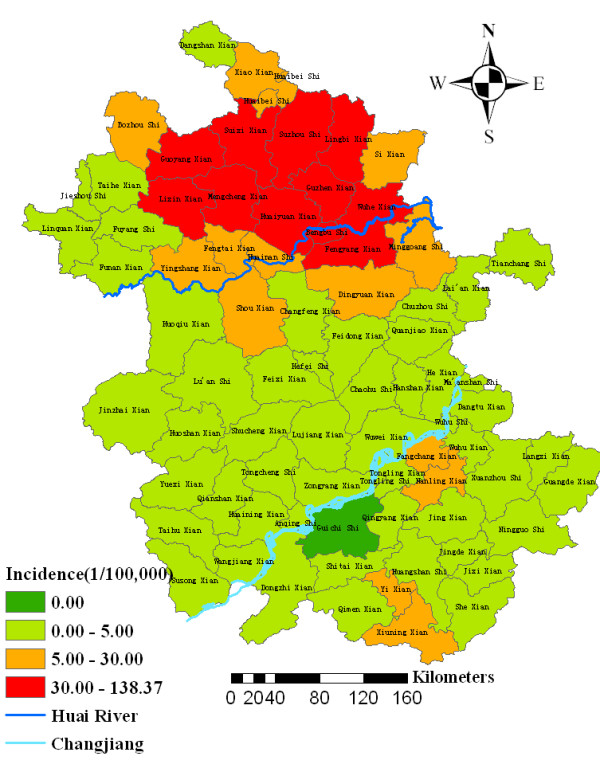
Annualized average incidence of malaria in Anhui province, China, 2000–2006.

In Anhui Province, a total of 77,674 malaria cases had been reported from 2000 to 2006. Annualized average incidence at the county-level ranged from 0 to 138.37 per 100,000. Among the total 78 counties and cities in Anhui Province, one county was non-endemic (covering 1.89% of total land and occupied by 0.95% of the total population), 52 counties and cities were low-endemic (covering 66.62% of total land and occupied by 61.40% of the total population), 14 counties and cities were medium-endemic (covering 17.57% of total land and occupied by 17.96% of the total population), and 11 counties and cities were high-endemic (covering 13.92% of total land and occupied by 19.69% of the total population). Most of the cases in Anhui Province occurred on low-lying lands along the Huai River. The four type areas were displayed in the thematic map as showed in Figure 1.

**Figure 2:**
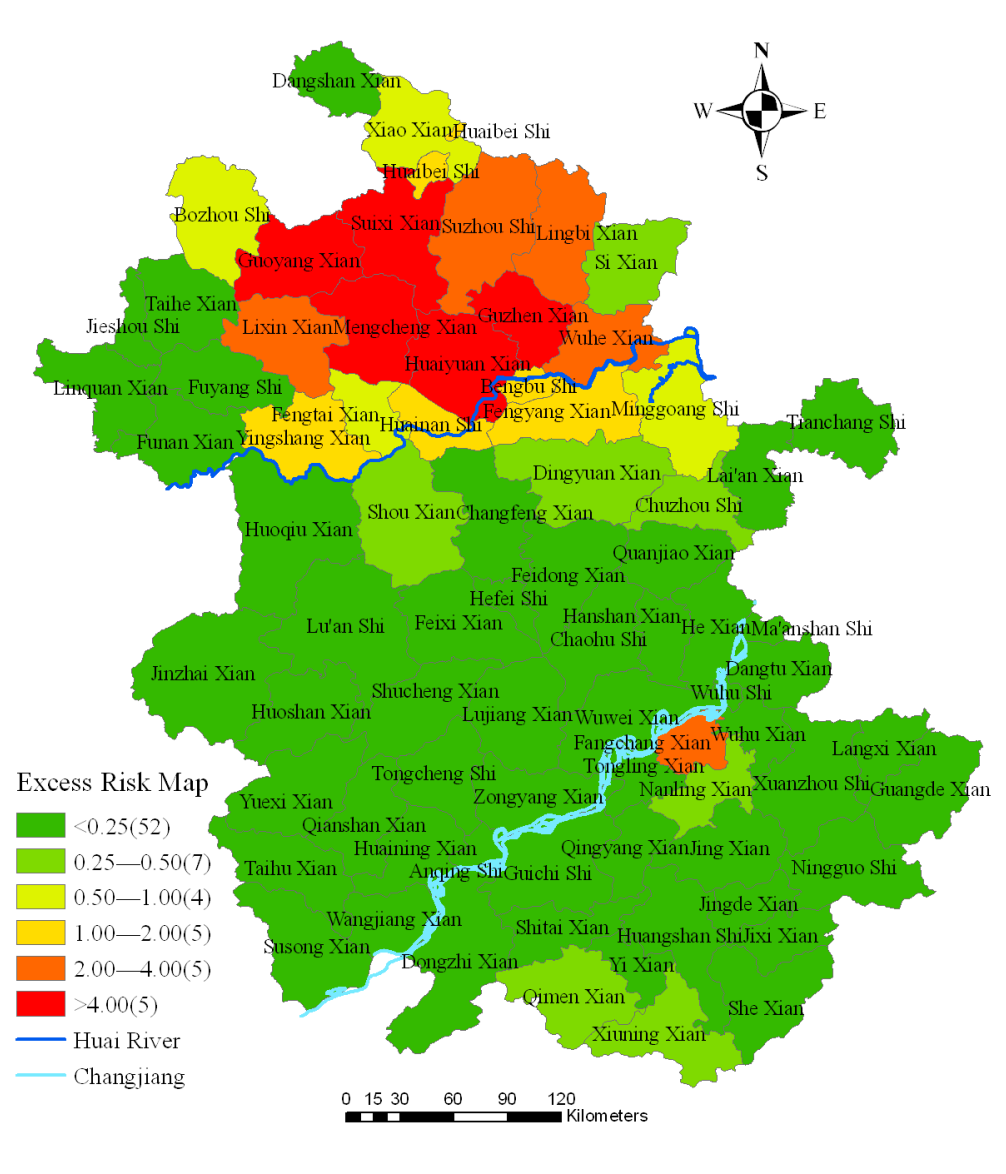
Excess risk map of malaria in Anhui province, China, 2000–2006.

The excess hazard map showed distribution of the excess risk, which was defined as a ratio of the observed number over the expected number of cases. Counties in dark green color had lower incidences than expected, as indicated by excess risk values less than 1. In contrast, counties in red color had incidences higher than expected or excess risk values greater than 1 (Figure 2). The excess risk is a non-spatial measure, which ignores the influence of spatial autocorrelation.

**Figure 3:**
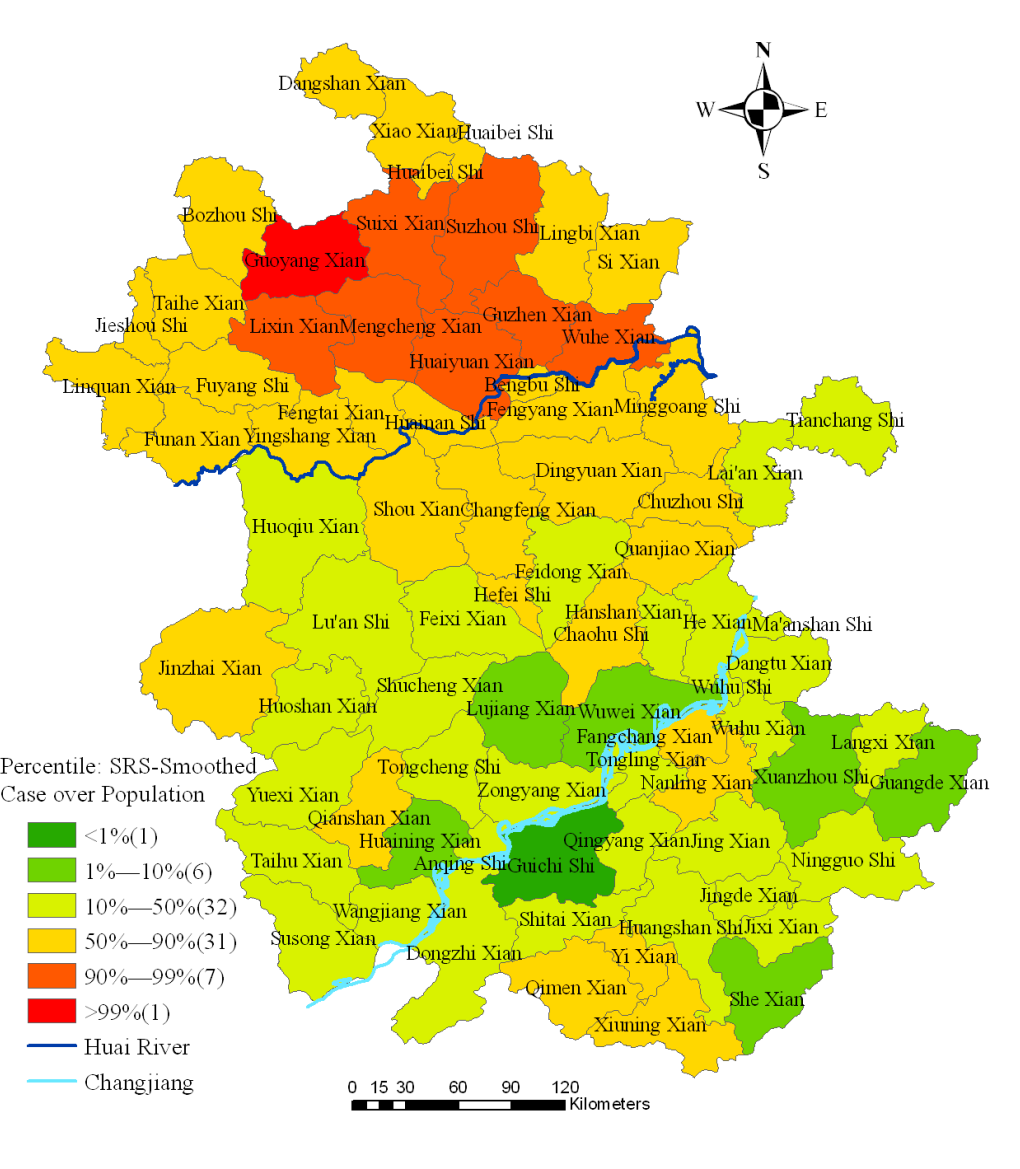
Spatially smoothed percentile map of malaria in Anhui province, China, 2000–2006.

Spatially smoothed percentile map for annualized average incidence was created for correcting the variance instability of incidences, and six neighbours identified for each county by k-nearest neighbours criterion provided the most appropriate map of smoothed incidences (Figure 3).

### Spatial clustering of malaria in Anhui Province

**Figure 4:**
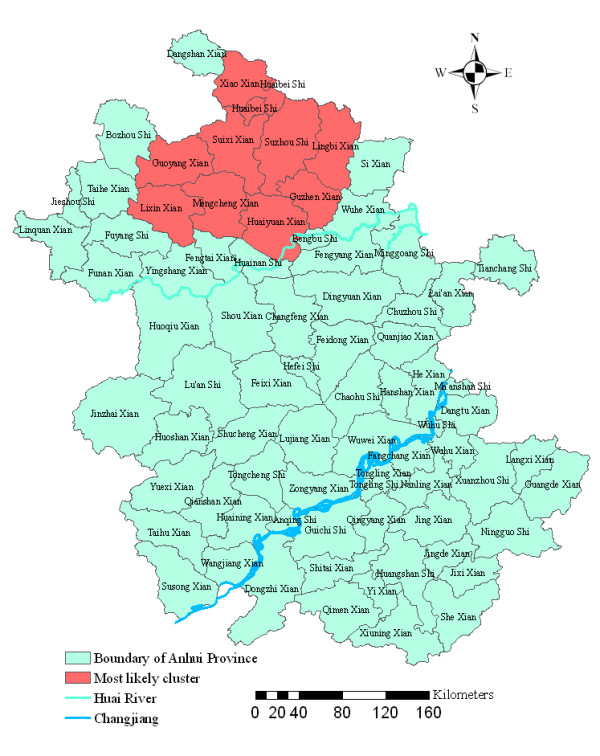
Spatial distribution of clusters of malaria with significant higher incidence using the maximum cluster size < 50% of the total population in Anhui province, China, 2000–2006.

Analysis of cases of malaria in 2000–2006 in Anhui Province showed that malaria was not distributed randomly. Using the maximum spatial cluster size of < 50% of the total population, the spatial cluster analysis identified a most likely cluster that included eight counties and two cities, which all located in the north of Huai River (Figure 4). The identified cluster contained 8.31% of the area's total population. The overall RR within the cluster was 39.75, with an observed number of cases of 43,182 compared with 2,443 expected cases. This elevated risk within a non-random pattern of disease distribution was significant (*P *< 0.001) (Table 1).

**Table 1:** SaTScan statistics for the most likely cluster, Anhui province, China, 2000–2006.


			Secondary clusters			
			
SaTScan statistics		Most Likely cluster	2	3	4	5

The 1^st ^iteration^1^	Observed^a^	43182				
	Expected^b^	2443				
	RR^c^	39.746				
	*P*-value	0.001				
	Cluster radius (km)	79.50				
	Observed^a^	43182	7	2890	281	60
The 2^st ^iteration^2^	Expected^b^	2443	0.39	2245	208	40
	RR^c^	39.746	18.068	1.299	1.351	1.504
	*P*-value	0.001	0.001	0.001	0.001	0.794
	Cluster radius (km)	79.50	0^d^	102.61	0^d^	0^d^

a: The number of observed cases in a cluster.

b: The number of expected cases in a cluster.

c: Relative risk.

d: Only 1 county was included in the cluster.

1: Maxmum cluster size set at 50% of the study population.

2: Maxmum cluster size set at 25% of the study population.

**Figure 5:**
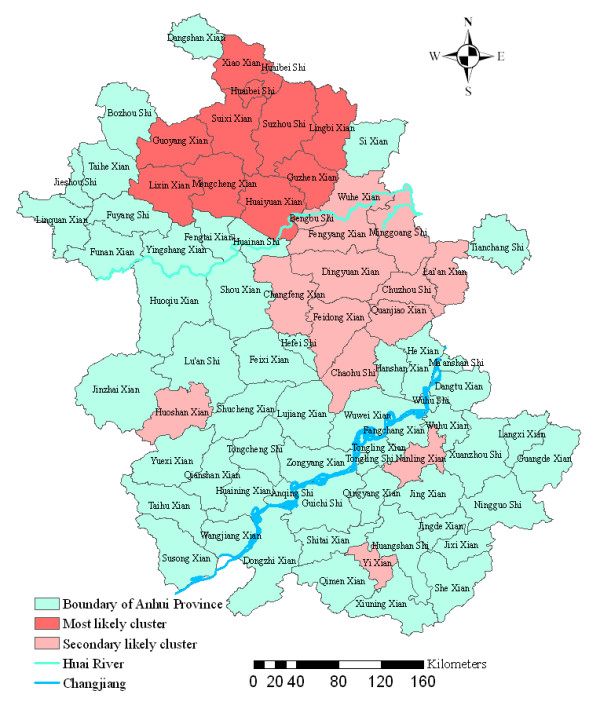
Spatial distribution of clusters of malaria with significant higher incidence using the maximum cluster size < 25% of the total population in Anhui province, China, 2000–2006.

To investigate the possibility of smaller clusters, the same analysis was performed with a modification of the maximum spatial cluster size which was defined as < 25% total population. A most likely cluster and four secondary clusters were identified (Figure 5). The most likely cluster was the same as in the 50% analysis. Four secondary sub-clusters included 14 counties which contain 8.39% of the total population. This excess risk within a nonrandom distribution pattern except one sub-cluster was also significant (*P *< 0.001) (Table 1).

## Discussion

The investigation of infectious disease clustering is receiving renewed interest, not least because of advances in geographical information systems (GIS) and spatial statistics, which allow for the quantification of the degree of clustering of infections. Such approaches have been used to investigate the spatial clustering of dengue [16], sleeping sickness [17], human granulocytic ehrlichiosis [12], haemorrhagic fever with renal syndrome [18], but their application to malaria has been limited [19,20]. An improved understanding of the spatial clustering of malaria on low-lying lands in China may provide useful insights into local epidemic control and resource allocation.

Using GIS and spatial statistics, the spatial distribution of confirmed malaria cases and increased risk regions in the highly endemic area were identified. The spatial statistics analyses clearly yielded a nonrandom distribution of malaria in the province. Spatial smoothing identified areas of increased risk located in the north of Huai River, the third largest river in China (Figure 3). Spatial cluster analysis identified a statistically significant cluster in the same area, in the north of Huai River, including the counties and cities of Suixi, Guoyang, Xiao, Mengcheng, Guzhen, Lingbi, Huaiyuan, Lixin, Suzhou and Huaibei, where *Anopheles sinensis *is the principal vector and the people have the habit of sleeping in the open in the summer [21]. These areas were different from the previous foci in the centre part of the province during the past two decades, where *Anopheles anthropophagus *is the principal vector [21].

On the basis of this study, prevention strategies are recommended that focus on these high epidemic areas. In an area where malaria disease is highly endemic, targeting prevention strategies at areas of highest risk can potentially increase the programme's effectiveness. People at highest risk should be informed of the high risk and the possibilities for risk reduction. Funds spent on programmes might be better spent on areas where cost-effectiveness can be maximized.

The present study analysed the associations between human population and malaria cases only. Gathering and including vector population data (including species, population density, distribution and infection prevalence rates) and environmental variables in the risk analysis of malaria in these areas provide a more comprehensive view of the disease risk. Future research, to investigate the underlying causes of increased risk in the identified areas, will be analysis of landscape attributes and identification of the environmental variables characteristic of high-risk areas.

## Conclusion

This study analysed the spatial distribution of malaria in Anhui Province, China, during 2000 to 2006 using the spatial smoothing and spatial scan statistic method. SaTScan identified a geographic area in northern Anhui Province as the most likely endemic cluster region. This has not been previously reported. The data show practical malaria control measures, as well as methods for future study of malaria and other vector-borne diseases. Further, GIS and GIS-based spatial statistical techniques may provide an opportunity to clarify and quantify the epidemic situation of malaria within re-emerged epidemic areas, and lay a foundation to pursue future investigations into the environmental factors responsible for the increased disease risk. To implement specific and geographically appropriate risk-reduction programmes, the use of such spatial analysis tools should become an integral component in epidemiology research and risk assessment of malaria.

## Competing interests

The authors declare that they have no competing interests.

## Authors' contributions

WZ, LW, YX and SL were involved in the conceptualization, research design, execution and write-up of the first draft of the manuscript. LF, JM, FH and JW contributed to database design and data analysis, JJ, LF, HY and WC all advised on the design of the study, and the analysis and interpretation of the results. All authors were involved in the preparation of the manuscript.
